# In Vitro Antibacterial and Anti-Inflammatory Effects of Novel Insect Fungus *Polycephalomyces phaothaiensis* Extract and Its Constituents against *Propionibacterium acnes*

**DOI:** 10.3390/antibiotics9050274

**Published:** 2020-05-25

**Authors:** Witsanu Sonyot, Supaporn Lamlertthon, Janet Jennifer Luangsa-ard, Suchada Mongkolsamrit, Kanchana Usuwanthim, Kornkanok Ingkaninan, Neti Waranuch, Nungruthai Suphrom

**Affiliations:** 1 Department of Chemistry, Faculty of Science, Naresuan University, Phitsanulok 65000, Thailand; witsanu_sy@hotmail.co.th; 2 Centre of Excellence in Fungal Research, Department of Microbiology and Parasitology, Faculty of Medical Science, Naresuan University, Phitsanulok, 65000, Thailand; paolamlertthon@gmail.com; 3 Plant Microbe Interaction Research Team, BIOTEC, 113 Thailand Science Park, Pathum Thani 12120, Thailand; jajen@biotec.or.th (J.J.L.-a.); suchada@biotec.or.th (S.M.); 4 Cellular and Molecular Immunology Research Unit, Faculty of Allied Health Sciences, Naresuan University, Phitsanulok 65000, Thailand; kanchanau@nu.ac.th; 5 Bioscreening Unit, Department of Pharmaceutical Chemistry and Pharmacognosy, Faculty of Pharmaceutical Sciences and Center of Excellence for Innovation in Chemistry, Naresuan University, Phitsanulok 65000, Thailand; k_ingkaninan@yahoo.com; 6 Faculty of Pharmacy, Airlangga University, Surabaya 60286, Indonesia; 7 Cosmetics and Natural Products Research Center, Department of Pharmaceutical Technology, Faculty of Pharmaceutical Sciences and Center of Excellence for Innovation in Chemistry, Naresuan University, Phitsanulok 65000, Thailand; netiw@nu.ac.th; 8 Department of Chemistry, Faculty of Science and Center of Excellence for Innovation in Chemistry, Naresuan University, Phitsanulok 65000, Thailand

**Keywords:** *Polycephalomyces phaothaiensis*, entomopathogenic fungi, *Propionibacterium acnes*, anti-inflammation, cytokines

## Abstract

*Propionibacterium acnes* plays an important role in the pathophysiology of acne vulgaris, the most common chronic inflammatory skin disease of the pilosebaceous unit. This study was conducted to investigate whether the entomopathogenic fungus *Polycephalomyces phaothaiensis* components have antibacterial and anti-inflammatory effects against *P. acnes* that may serve for acne treatment. A chemical study by spectroscopic analysis resulted in the identification of seven known compounds. The anti-*P. acnes* potency of extracts and test compounds was determined by both agar diffusion and broth dilution methods. The ethyl acetate extract from culture broth along with cordytropolone (**1**) and stipitalide (**2**) exhibited strong anti- *P. acnes* activity while (+)-piliformic acid (**3**) showed weak inhibitory activity. The anti-inflammatory effect of ethyl acetate extract and **1**–**3** was then examined by the quantification of pro-inflammatory cytokines IL-1β, IL-6, and TNF-α on heat-killed *P. acnes* induced cytokine production by THP-1 cells. The result demonstrated that the extract and its constituents (**1**–**3**) showed a potent significant effect by inhibiting the *P. acnes*-induced pro-inflammatory cytokines production in THP-1. Our results suggest for the first time that *P. phaothaiensis* and its constituents (**1** and **2**) hold therapeutic value for further studies as a new alternative treatment for acne.

## 1. Introduction

Acne vulgaris is a common chronic inflammatory skin disease affecting 90% of all global teenagers. It causes dermatological problems of the pilosebaceous unit located on the face, chest, back, and other body parts [1] and may also result in a high degree of a psychological burden as well [2]. It was reported that emotional stress caused by acne is equivalent to that of diabetes and epilepsy [3]. Currently, the main purpose of acne treatment is to control the inflammatory reaction caused by excessive sebum secretion, hyperkeratosis, and *Propionibacterium acnes* (*P. acnes*) proliferation.

Acne occurs mainly in adolescence when, due to hormonal imbalance, an oily substance is increasingly secreted by the sebaceous glands (influenced by androgens) to induce hyperkeratosis of the follicular wall, causing the formation of microcomedones. Additionally, keratinocyte proliferation in comedones is increased, leading to sebum-blocked skin hair follicles. Under anaerobic conditions, the proliferation of *P. acnes* activates monocytic cell immune responses of the host to induce the expression of pro-inflammatory cytokines such as tumour necrosis factor (TNF)-α, interleukin (IL)-6, and interleukin (IL)-1β [4]. In some conditions, *Staphylococcus aureus*, a skin commensal organism, can become an opportunistic pathogen causing inflammatory skin diseases such as pustules, furuncles, abscesses, and folliculitis [5]. This organism also causes secondary infection of the pilosebaceous unit following *P. acnes* overgrowth, leading to more severe acnes.

The current topical effective drug to use for inflammatory or non-inflammatory lesions are retinoids which inhibit comedone formation and inflammation. The treatment can be as a topical anti-acne preparation and oral antibiotics, and they are either used alone or in combination with steroids to dampen the inflammatory response, or benzoyl peroxide, which has an antibacterial effect. Isotretinoin is also used for severe acne because of its anti-inflammatory effect through controlling hyperkeratosis, sebum secretion, and *P. acnes* [6]. However, the use of topical retinoids may induce skin irritation, erythema, and scaling. Use of isotretinoin requires caution as there is the documented side effect of birth defects and embryopathy for pregnant mothers after their exposure to isotretinoin [7]. Continuous use of oral antibiotics may also lead to resistance to bacteria, as *P. acnes* resistance toward erythromycin (50%), clindamycin (35%), and tetracycline (25%) has been reported [8]. Therefore, the development of additional remedies and the search for possibly natural ingredients for the treatment of acne as a substitute are of particular importance. Natural resources have been investigated for a long time due to their high chemical diversity and unique pharmacological effects. Entomopathogenic fungi have been considered as one potential source of natural bioactive substances and demonstrated potent biological activity, including antibacterial and anti-inflammatory effects [9].

Recently, the novel hyperparasitic fungus of *Ophiocordyceps* species was discovered in Ban Phaothai community forest, Phitsanulok province, Thailand. It was identified as *Polycephalomyces phaothaiensis* [10]. From the preliminary study, the crude extracts of *P. phaothaiensis* showed high antibacterial activity against *P. acnes*. Interestingly, bioactivities of this fungus have not yet been reported. The purpose of the current study is to isolate and identify the anti-*P. acnes* and anti-inflammatory constituents.

## 2. Results and Discussion

**Figure 1:**
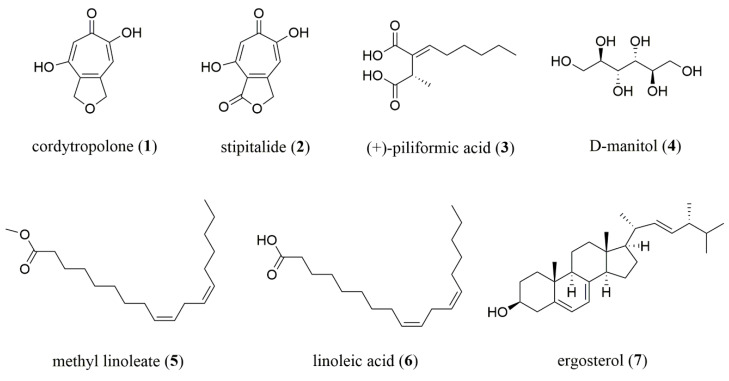
Structures of compounds **1**–**7** isolated from *P. phaothaiensis.*

Both culture medium and mycelia of novel insect fungus, *P. phaothaiensis*, after cultivation in potato dextrose agar broth (PDB) for 14 days were extracted to give four crude extracts. Chromatographic fractionation and purification of extracts resulted in the isolation of two known tropolone derivatives, namely cordytropolone (**1**) [11] and stipitalide (**2**) [12], along with five known compounds, namely (+)-piliformic acid (**3**) [13], d-manitol (**4**), methyl linoleate (**5**), linoleic acid (**6**), and ergosterol (**7**). Their structures (Figure 1) were elucidated on the basis of spectroscopic data, which are given in Supplementary Tables S1 and S2 and Figures S1–S15, and their spectra were also compared with reported data [14,15,16]. Based on the literature review, compounds **1** and **2** have been reported as the metabolites in some fungi [11,17,18]. Compound **3** has been isolated in several fungi of Xylariaceous genera [19]. In addition, compounds **4**–**6**, which are the constituents, could be found in natural products and the artificial cultivation of fungi was also present in other entomopathogenic fungi [9]. Compound **4** is widely recognized polyol in fungi, where it is found in spores, fruiting bodies, and mycelia. It has also been ascribed a multitude of roles in filamentous fungi, including carbon storage from nutrient source, reservoir of reducing power, stress tolerance, and spore dislodgement and/or dispersal [20,21]. Moreover, compound **4** formation in fungi could be raised from the metabolism of dextrose from the cultivation broth [22]. Although compound **5**, a fatty acid methyl ester of **6**, was also isolated from our sample, the possible artefact from the isolation procedure where the methanol was used should be noted and concerned. Ergosterol (**7**), which is a sterol commonly found in cell membranes of fungi was also observed in our study. Herein, it is noted that the present study describes the isolation and identification of chemical constituents from *P. phaothaiensis* for the first time.

In order to assess the antibacterial activity of *P. phaothaiensis* against *P. acnes*, a disc-diffusion assay was conducted with four crude extracts (2 mg/disc). Their growth inhibitory activity is shown in Table 1. Among the four tested extracts, the ethyl acetate extract (EPP) obtained from the culture medium expressed the highest inhibitory activity against *P. acnes* (inhibition zone of 47.2 ± 0.8 mm). A similar growth inhibition profile was observed with the positive control, clindamycin (2 μg/disc) while methanolic broth extract (MBPP) was inactive. In addition, two mycelial extracts, namely dichloromethane extract (DPP) and methanolic extract (MPP), were found to be moderately effective in inhibiting with the inhibition zone of 13.8 ± 0.2 mm and 24.8 ± 0.2 mm, respectively. Furthermore, three active extracts were further investigated by determination of their minimum inhibitory concentration (MIC) and minimum bactericidal concentrations (MBC).

As a result, the EPP extract inhibited the growth of *P. acnes* with the lowest a MIC of 16 µg/mL and MBC values at 32 µg/mL. The MIC of clindamycin was 1 µg/mL for *P. acnes* DMST 14916 (Table 1). Thus, only the EPP extract was investigated further to isolate the anti-*P. acnes* constituents. Three isolated compounds from the potent extract (EPP), compounds **1**–**3**, were tested for their antibacterial activity against *P. acnes*. Among the three tested compounds, **1** and **2** expressed potent inhibitory activity against *P. acnes* with the inhibition diameter zone (250 µg/disc) of 46.8 ± 0.2 mm and 45.0 ± 0.8 mm, but this effect was not statistically significant. As a result of MIC and MBC measurements (Table 1 and Figure S16), it was revealed that compound **1** at 8 µg/mL and 16 µg/mL had the most potent anti-*P. acnes* property among the other compounds and extracts tested. Interestingly, **2** also expressed a similar trend to the MIC (64 µg/mL) and MBC (128 µg/mL) values compared to compound **1**. In addition, compound **3** demonstrated low activity on *P. acnes*.

Based on the structures of **1** and **2**, they were tropolone derivatives. Compound **1** was composed of a cyclic ether (tetrahydrofuran) fused with tropolone ring while **2** was composed of a cyclic ester (γ-lactone ring). From our results, it might be possible that the presence of γ-butyrolactone in **2** may be hydrolyzed by *P. acnes* lipase which an enzymatic-catalyzed ring-opening hydrolysis of lactones [23,24] and led to a decreased potency of anti-*P. acnes* activity (in terms of MIC and MBC values). Moreover, other factors might have been involved in the activity, which needs to be further investigated.

**Figure 2:**
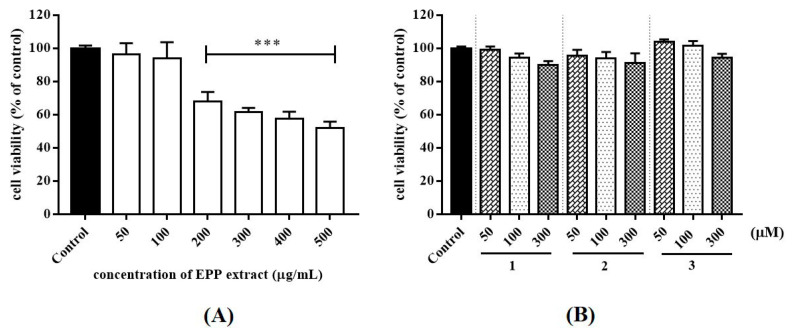
Cell viability on THP-1 cells after treatment for 24 h with the EPP extract (**A**), compounds **1**–**3** (**B**). Cell viability was determined by MTT assay. Results represent as mean ± SEM in triplicate experiment. 1% DMSO was use as a control group. *** *p* < 0.001 vs. control.

Acne is a chronic skin disease caused by inflammation, elevated levels of skin sebum, and growth of *P. acnes*. Moreover, *P. acnes* promotes inflammation by inducing IL-1β, IL-6, and TNF-α production on monocytes. Monocytes belong to the innate immune compartment, in which their major roles are recognition of foreign pathogens such as bacteria. THP-1 cells resemble primary monocytes and was used as an in vitro cell model for immune modulation approach [25]. Flow cytometry was used for examining alterations to monocytes, in which THP-1 cell was characterized by their level of cluster of differentiation (CD)14 and CD16 expression [26]. In this study, THP-1 cell was used to: (i) assess the cytotoxicity of test samples and (ii) find the suitable concentrations to further assess heat-killed *P. acnes*-stimulated inflammatory cytokines production. Cell viability after treatment with various concentrations of samples was determined by MTT assay. The concentration of compounds was limited by the solubility in culture medium. EPP (50 and 100 µg/mL) and compounds (50, 100 and 300 µM) presented favorable cell survival rate of over 90% (Figure 2). Accordingly, this concentration for the levels of cytotoxicity was generally low.

**Figure 3:**
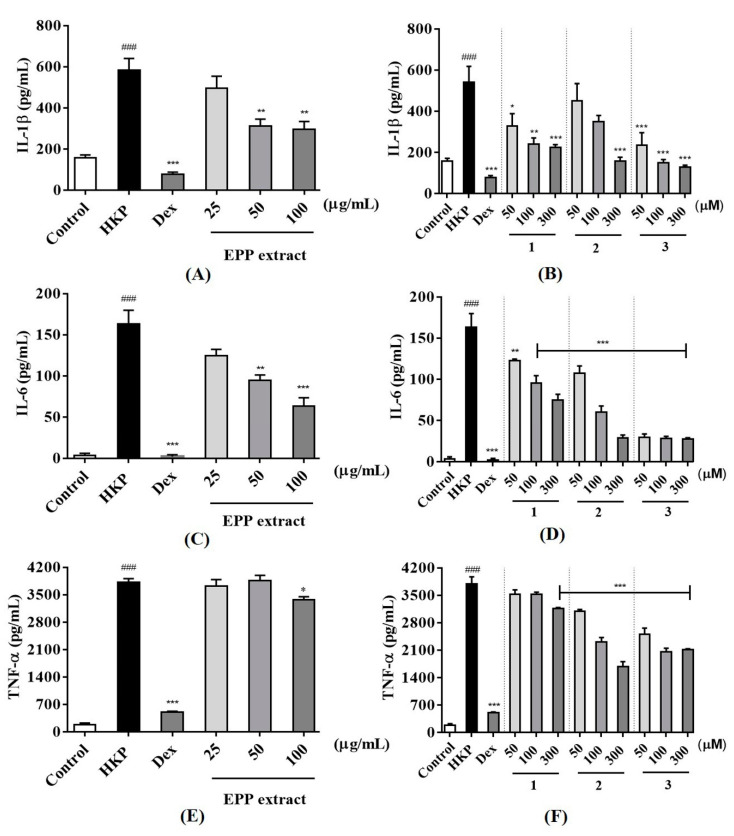
Effect of the EPP extract, compounds **1**–**3** on pro-inflammation cytokines production in THP-1 cells. THP-1 cells were stimulated with heat-killed *P. acnes* (HKP) 1 × 10^7^ CFU/mL and then incubated with the indicated doses of EPP extract (25, 50 and 100 μg/mL), compounds **1**–**3** (50, 100 and 300 μM) and Dexamethasone (Dex, 1 μM). After 24 h, the supernatants were harvested for measuring pro-inflammatory cytokines concentration, including IL-1β (**A** and **B**), IL-6 (**C** and **D**) and TNF-α (**E** and **F**) by ELISA kit. The data were expressed as mean ± SEM in triplicate experiment; ^###^ *p* < 0.001 vs. control, * *p* < 0.05 vs. HKP, ** *p* < 0.01 vs. HKP, *** *p* < 0.001 vs. HKP.

For the quantification of inflammatory cytokines from heat-killed *P. acnes* stimulated THP-1 cell, cytokines measurement was made by ELISA kit. As a result, heat-killed *P. acnes* -stimulated THP-1 significantly increased IL-1β, IL-6, and TNF-α production (Figure 3). None of samples from *P. phaothaiensis* gave a higher cytokines reduction over the positive drug control, dexamethasone (1 µM). However, the treatment with EPP extract showed a significant effect in cytokines reduction. Furthermore, compounds **1**–**3** tended to decrease IL-1β, IL-6, and TNF-α level of inflamed cell. In acne research, *P. acnes* might trigger pro-inflammatory cytokine response in acne via stimulation of the toll-like receptor 2 (TLR2), playing a critical role in the innate immunological response to a variety of microbial pathogens [27]. *P. acnes* is able to directly induce the production of pro-inflammatory cytokines (IL-1β, IL-6, and TNF-α) by monocytic cells [28]. Among them, IL-1β mediates the local inflammatory reaction of white blood cells. It plays an important role in acnes and skin infection as inflammation generated at the sebaceous gland by *P. acnes* is highly dependent upon IL-1β [29].

Our results showed that EPP and its active compounds suppressed pro-inflammatory cytokines IL-1β, IL-6, and TNF-α production of heat-killed *P. acnes* induced THP-1. Moreover, 50 and 100 μg/mL of EPP, 50–300 μM of **1** and **3**, and only 300 μM of **2**, significantly reduced IL-1β level compared to heat-killed *P. acnes* stimulated THP-1 (Figure 3A,B). IL-6 and TNF-α are also increased in acnes lesion and are secreted by *P. acnes* [4]. As evidenced from Figure 3, it was found that 50 and 100 μg/mL of EPP and 50–300 μM of all active compounds effectively suppressed IL-6 in inflamed cells (Figure 3C,D). Only 100 μg/mL of EPP, 300 μM of **1**, and 50–300 μM of **2** and **3** significantly decreased heat-killed *P. acnes* induced TNF-α activation (Figure 3E,F). However, when the pro-inflammatory cytokines are secreted due to *P. acnes*, arachidonic acid is also produced from phospholipids of the cell, and it causes an inflammatory reaction as it is converted to the pro-inflammatory prostaglandins by the action of cyclooxygenase-2 (COX-2) [30].

From the data, two tropolone compounds (**1** and **2**) could reduce the concentrations of pro-inflammatory cytokines in heat-killed *P. acnes* stimulated THP-1. The ability of these group of compounds to exert anti-inflammatory effect corresponded to a study by Shih et al. [31]. They demonstrated that β-thujaplicin, a tropolone compound with a seven-membered carbon ring and an isopropyl side chain, exhibited dose-dependent inhibition on PGE2, IL-6, and TNF-α production, as well as iNOS, COX2, and NF-kB protein expression. In agreement with in vitro studies, β-thujaplicin effectively inhibited LPS-induced NO and TNF-α production and caused a significant decrease in the mortality rate of mice suffering from septic shock [31]. The results of **1** and **2** suggested that tropolone compounds containing a cyclic ester (lactone ring) in **2** instead of cyclic ether (tetrahydrofuran) in **1** led to the improvement of pro-inflammatory cytokines reduction. However, other factors might have been involved in this activity. As seen in compound **3**, changing the basic skeleton to dicarboxylic acid could increase the inhibition of IL-1β and IL-6. However, it should be noted that our result suggests the potential of EPP extract *P. phaothaiensis* and its active compounds (**1**–**3**) to reduce the levels of IL-1β, IL-6, and TNF-α in heat-killed *P. acnes* induced THP-1.

## 3. Materials and Methods

### 3.1. General Experimental Procedures

A silica gel column (0.040–0.063 mm granule size) and sephadex LH-20 were used for the chromatographic isolation of the extract components. Thin layer chromatography (TLC) analysis was performed on TLC silica gel 60 F254 aluminum sheet 20 × 20 cm (Merck, Darmstadt, Germany). Nuclear magnetic resonance (NMR) spectra were recorded on a Bruker AV400 (USA) spectrometer at 400 MHz for proton and 100 MHz for carbon. Moreover, an Agilent 1260 infinity high performance liquid chromatography instrument via an ESI interface was connected to a 6540 ultrahigh definition accurate mass Q-TOF (Agilent Technologies, Palo Alto, CA, USA). Fourier-transform infrared spectrophotometer (FT-IR), PerkinElmer Spectrum GX (PerkinElmer, Waltham, MA, USA). The absorbance was measured using hybrid Multi-Mode Detection Synergy H1 (Model H1MF) (Bio-TeK Instruments, Winooski, VT, USA). In addition, a McFarland tube densitometer (Grant Instruments DEN-1B, Beaver Falls, PA, USA) and semiautomatic fermentor-biorector–MDFT –N—5 L with mono mode controller (BE Marubishi, Japan) were used.

### 3.2. Chemicals

The sterile filter paper discs (Macherey-Nagel, MN^®^, Germany), clindamycin (2 μg/disc) by Oxoid (Basingstoke, UK) were used for antibacterial assay. The Brain heart infusion broth, Müller-Hinton broth and agar powder were bought from HiMedia Laboratories Pvt. Ltd. (Mumbai, India). Gas generation kit (AnaeroPack-Anaero) from MITSUBISHI Gas chemical (Tokyo, Japan). 3-(4,5-dimethylthiazol-2-yl)-2,5-diphenyltetrazolium bromide (MTT) and phorbol-12-myristate-13-acetate (PMA) was purchased from Sigma-Aldrich (Sigma Aldrich, Missouri, MO, USA). RPMI-1640 medium, supplemented with 10% fetal bovine serum (FBS) and 1% antibiotic mixture of penicillin G (10,000 units/mL)-Streptomycin (10,000 μg/mL) (Thermo Fisher Scientific, Inc., New York, NY, USA). In addition, analytical grade methanol, 95% ethanol, ethyl acetate, dichloromethane, dimethyl sulfoxide and hexane from RCI Labscan Ltd. (Bangkok, Thailand) were used. A Sandwich ELISA kit was used for pro-inflammatory cytokines detection following the manufacturer’s protocol (BioLegend, Inc., California, CA, USA).

### 3.3. Organism and Sample Collection

*Polycephalomyces phaothaiensis* BCC78485 was obtained from the BIOTEC culture collection (BCC). The fungus was collected on 25 September 2015 at Ban Phaothai community forest, Tambon Chomphu, Noen Maprang District, Phitsanulok Province, Thailand. It was isolated and cultured on potato dextrose agar broth (PDB) and a living culture is maintained at BCC.

### 3.4. Culture Condition and Fungal Extraction

The fungal culture used throughout the experiment was maintained on potato dextrose agar (PDA) slants at 25 °C. For inoculum preparation, the fungus was initially grown at 25 °C on a PDA plate for 7 days. A 0.7 cm^2^ plug from the outer zone of the colony was punched with a sterile cutter and transferred to 300 mL PDB in a 500 mL flask and grown at 27 °C at 170 rpm under on a rotary shaker for 14 days. The culture was then transferred to a 3 L-fermenter of PDB for mycelial production using another 14 days (27 °C, 170 rpm). After that, the culture mycelia and culture broth were separated by sterile cloth to give mycelia biomass (457 g) and culture broth (1.5 L). The culture broth was extracted by maceration with ethyl acetate (1 L × 3 times) for 24 h at room temperature. The organic layer was pooled and evaporated under reduced pressure to produce the ethyl acetate extract (EPP, 758 mg). The residual culture broth (an aqueous layer) was freeze-dried and methanol was then added to give methanol soluble portion. This portion was evaporated and labeled as methanolic broth extract (MBPP, 14.4 g). For the mycelia extraction, the sequential extraction with organic solvents were used. The extraction was started by the extraction of mycelia with methanol (1 L) for 5 days (for three times) at room temperature to give methanolic extract (MPP, 7.70 g). The residual mycelial was further extracted with dichloromethane (1 L × 3 times) to give dichloromethane extract (DPP, 513 mg). All of these extracts were kept under –20 °C before use.

### 3.5. Isolation and Identification of Isolated Compounds

The EPP extract (558 mg) was chilled twice in each of the following solvents; ethyl acetate (15 mL), absolute ethanol (15 mL) and deionized water (5 mL). During the process, compound **1** (141 mg) precipitated as a colorless solid. The remainder of EPP extract (400 mg) was further fractionated on a Sephadex LH-20 column (2 × 93 cm) using methanol (2L) as a mobile phase to yield five fractions (S1–S5). Fraction S3 (117 mg) was further fractionated on a Sephadex LH-20 column (1.5 × 90 cm) and eluted with methanol to give three fractions (S3/1–S3/3). Fraction S3/2 (35.6 mg) was re-fractionated using Sephadex LH-20 column to give compound **2** (4.5 mg) as a reddish-brown amorphous powder. Fraction S4 (28.5 mg) was chromatographed on a silica gel column and a gradient sequentially formed of *n*-hexane and dichloromethane as the mobile phase to yield white solid of **3** (10 mg).

Furthermore, the MBPP (12.0 g) and MPP (1.0 g) extracts were washed by adding methanol (200 mL × 3 times) to provide compound **4** (1.02 g). Moreover, the DPP extract (370 mg) was chromatographed by silica gel column chromatography and gradient sequential elution with the mixtures of *n*-hexane-ethyl acetate and dichloromethane-methanol were performed successively. The eluent was collected in 5 mL fractions to yield 8 fractions (DPP1-8). Compound **5** (10 mg) was obtained from fraction DPP2, which eluted with gradient ratio of petroleum ether-dichloromethane. Fraction DPP4 (30 mg) was subjected to silica gel column chromatography and eluted with gradient ratio of hexane and ethyl acetate to give **6** (20 mg). Finally, fraction DPP5 (102 mg) was washed with methanol (10 mL × 3 times) and re-precipitated with ethyl acetate at room temperature to provide **7** (38 mg). The structures of all isolated compounds were elucidated using NMR, MS, and IR. Their structures were also confirmed by comparison of their spectral data with reported data.

Cordytropolone (**1**): colorless solid, C_9_H_8_O_4_, ESI-MS (neg. ion mode) *m/z* 179.037 [M - H]^−^; FT-IR (ATR) ν_max_ (cm^−1^): 3188, 1603, 1520, 1434, 1377, 1275, 1219, 1171, 917, 762, 710.

Stipitalide (**2**): reddish-brown amorphous, C_9_H_7_O_5_, ESI-MS (pos. ion mode) *m/z* 195.0292 [M + H]^+^, FT-IR (ATR) ν_max_ (cm^−1^): 3146, 1716, 1628, 1482, 1228, 1168, 1048, 1028.

(+)-Piliformic acid (**3**): white solid, C_11_H_18_O_4_; ESI-MS (neg. ion mode) *m/z* 213.119 [M - H]^−^; ${\left[\alpha \right]}_{D}^{28}$ +1.3 (MeOH, c 0.155); UV (MeOH) λ _max_ (log ε) 241 nm (2.67); FT-IR (ATR) ν_max_ (cm^−1^): 3250, 2926, 2857, 1684, 1626, 1410, 1292, 1256, 1223, 1058

d-mannitol (**4**): white solid, C_6_H_14_O_6_, ESI-MS (pos. ion mode) *m/z* 205.0679 [M + Na]^+^; FT-IR (ATR) ν_max_ (cm^−1^): 3234, 2875, 1635, 1411, 1363, 1062, 1032, 985.

Methyl linoleate (**5**): colorless oil, C_19_H_34_O_2_, EI-MS *m/z* 294 [M]^+^. Linoleic acid (**6**): colorless oil, C_18_H_32_O_2_, EI-MS *m/z* 280 [M]^+^. The identification of **5** and **6** were performed by computer matching their recorded mass spectra with a standard library; Wiley7n, and with the literature data.

Ergosterol (**7**): colorless solid, C_28_H_44_O, ESI-MS (pos. ion mode) *m/z* 396.65 [M + H]^+^; FT-IR (ATR) ν_max_ (cm^−1^): 3413, 2952, 2869, 1738, 1656, 1457, 1366, 1326, 1239, 1053, 1031, 967, 802.

### 3.6. Antibacterial Activity Assay

Four crude extracts and three isolated compounds (**1**–**3**) were assessed for antibacterial activity against *Propionibacterium acnes* DMST 14916. For bacterial culture preparation, the test organisms were cultured on brain heart infusion agar. *P. acnes* were incubated in an anaerobic environment at 37 °C for 72 h. After that, the fresh colonies was assessed and suspended to yield approximately 10^8^ CFU/mL.

The disc diffusion method was examined for antibacterial activity assay [32]. Briefly, a freshly prepared inoculum suspension was swabbed on the entire surface of brain heart infusion agar for *P. acnes*. The sterile filter paper discs (6.0 mm in diameter) were then loaded with 20 μL of each samples (2 mg/disc for extracts, 250 μg/disc for **1** and **2**, and 1 mg/disc for **3**), air-dried thoroughly, and aseptically placed on inoculated plate. Standard discs of clindamycin (2 μg/disc) was used as a positive control. The inoculated plates were then incubated at anaerobic environment. The results were recorded by measuring the diameters of growth inhibition zone (in mm). All experiments were performed in triplicate.

The minimum inhibitory concentration (MIC) and minimum bactericidal concentrations (MBC) of the potent samples (growth inhibition zone ≥ 9.5 mm) were determined by diluting to various concentrations. The assays were performed according to the methods described by CLSI and Balouiri et al. [32,33]. Various concentrations of tested samples were prepared in Müller-Hinton broth or brain heart infusion broth (contained 5% DMSO) in 96-well microtiter plate. After that, 10 μL of the bacterial suspensions were added into each well to give a final volume of 100 μL and the cell density corresponding to 10^5^ CFU/mL. The plates were then incubated at anaerobic environment for *P. acnes*. MIC was determined as the lowest concentration of test samples permitting no visible growth (no turbidity) when compared with the control tubes. Then, 10 μL of each culture broth was transferred on the agar plates and further incubated in an anaerobic environment. The lowest concentration with no visible bacterial growth on the agar surface was defined as the MBC.

### 3.7. Cell Viability Test

THP-1 were obtained from ATCC and cultured in completed media RPMI 1640, supplemented with 10% FBS and 1% Antibiotic-Antimycotic. Cell viability was determined using MTT assay. Cells were stimulated by 5 ng/mL PMA for 24 h in completed culture medium and seeded in 96-well plates at 3 × 10^4^ cell/well. The medium was then changed to serum free medium and tested samples (EPP extract and compounds **1**–**3**) for another 24 h. Then, the cells were washed once with phosphate-buffered saline (PBS) before 50 µL of 1 mg/mL of MTT reagent was added and incubated for 3 h. The medium was then removed and the formazan produced in viable cells was solubilized by adding 50 µL DMSO. The absorbance was measured at 595 nm using a microplate reader. The percentage of cell viability was calculated by comparing to the absorbance with control (non-treated) cells [34].

### 3.8. Preparation of Heat-Killed P. acnes

*P. acnes* (DMST 14916) was cultured on brain heart infusion agar and incubated in an anaerobic environment at 37 °C for 72 h. After that, the fresh colonies was assessed and suspended in 5 mL of PBS to yield approximately 10^8^ CFU/mL. The suspension of *P. acnes* was incubated at 80 °C for 30 min for the heat killing reaction. The heat killed *P. acnes* suspension was then stored at 4 °C until use [35].

### 3.9. Inflammatory Cytokines Measurement

To investigate the changes of pro-inflammatory cytokines, IL-1β, IL-6, and TNF-α levels were determined after heat-killed *P. acnes*—challenged THP-1 by sandwich ELISA kit. 2 × 10^5^ cells were seeded and stimulated by heat-killed *P. acnes* (1 × 10^7^ CFU/mL). Then, various concentrations of tested samples were added [25]. After 24 h, the supernatants were harvested for measuring pro-inflammation cytokines by human ELISA kit according to the manufacturer’s protocol. The production of cytokines in each condition were calculated from standard curves using known concentrations of recombinant cytokines.

### 3.10. Statistical Analysis

All experiments were performed in triplicate. Statistical comparisons were analyzed using one-way analysis of variance (ANOVA) with a multiple comparison method for data comparison among the experimental conditions.

## 4. Conclusions

The chemical constituents of a novel insect fungus, *P. phaothaiensis*, were identified and reported here for the first time. Moreover, this is the first study which reports the ability of *P. phaothaiensis* crude extract and its chemical constituents to inhibit *P. acnes* and exert an anti-inflammatory effect. The results suggested that *P. Phaothaiensis* and its active compounds (**1** and **2**) might be a new alternative source and marker compounds for the treatment of acne vulgaris.

## Figures and Tables

**Table 1:** Antibacterial effect of *P. phaothaiensis* extracts and isolated compounds against *P. acnes.*

Samples	Inhibition Diameter Zone (mm)	MIC (μg/mL)	MBC (μg/mL)
*Extracts*			
EPP	47.2 ± 0.8 ^a^	16	32
DPP	13.8 ± 0.2 ^a^	250	2000
MPP	24.8 ± 0.2 ^a^	500	4000
*Isolated compounds*			
cordytropolone (1)	46.8 ± 0.2 ^b^	8	16
stipitalide (2)	45.0 ± 0.8 ^b^	64	128
(+)-piliformic acid (3)	9.5 ± 0.5 ^c^	1000	4000
*Positive control*			
clindamycin	50.8 ± 0.6 ^d^	1	5

^a^ concentrations of extracts was tested using 2 mg/disc, ^b^ concentrations of **1** and **2** were tested using 250 μg/disc; ^c^ concentration of **3** was tested using 1 mg/disc, ^d^ concentration of clindamycin was tested using 2 μg/disc, EPP—ethyl acetate extract obtained from culture medium, DPP—dichloromethane extract obtained from mycelia, MPP—methanolic extract obtained from mycelia. The results of inhibition zone were recorded by measuring the diameters of growth inhibition zones (in mm) and expressed as mean ± SD. All experiments were done in triplicate.

## Supplementary Materials

The following are available online at https://www.mdpi.com/2079-6382/9/5/274/s1, Figure S1: Colony appearance and phylogenetic tree of *P. phaothaiensis* BCC78485, Figure S2: ^1^H-NMR spectrum of **1** (400 MHz, DMSO-*d_6_*), Figure S3: ^13^C-NMR spectrum of **1** (100 MHz, DMSO-*d_6_*), Figure S4: ^1^H-NMR spectrum of **2** (400 MHz, DMSO-*d_6_*), Figure S5: ^13^C-NMR spectrum of **2** (100 MHz, DMSO-*d_6_*), Figure S6: ESI-MS (pos. ion mode) spectrum of **2**, Figure S7: ^1^H-NMR spectrum of **3** (400 MHz, CDCl_3_), Figure S8: ^13^C-NMR spectrum of **3** (400 MHz, CDCl_3_), Figure S9: ^1^H-NMR spectrum of **4** (400 MHz, D_2_O), Figure S10: ^1^H-NMR spectrum of **5** (400 MHz, CDCl_3_), Figure S11: EI-MS spectrum of **5**, Figure S12: ^1^H-NMR spectrum of **6** (400 MHz, CDCl_3_), Figure S13: EI-MS spectrum of **6**, Figure S14: ^1^H-NMR spectrum of **7** (400 MHz, CDCl_3_), Figure S15: ^13^C-NMR spectrum of **7** (100 MHz, CDCl_3_), Figure S16: Minimum bactericidal concentration (MBC) test of extracts, cordytropolone (**1**), stipitalide (**2**), and (+)-piliformic acid (**3**) from *P. phaothaiensis* against *P. acnes* (**A**), *concentration at MIC values, Table S1: NMR data of **1** (in DMSO-*d_6_*) measured at 400 (^1^H) and 100 (^13^C) MHz, Table S2: NMR data of **3** (in CDCl_3_) measured at 400 (^1^H) and 100 (^13^C) MHz.
