# Supplementary material for: In Vitro Antibacterial and Anti-Inflammatory Effects of Novel Insect Fungus Polycephalomyces phaothaiensis Extract and Its Constituents against Propionibacterium acnes
Source: Antibiotics (Basel). 2020 May 25;9(5):274. doi: 10.3390/antibiotics9050274 (PMC7277416; doi:10.3390/antibiotics9050274)
Supplement: Supplementary file 1 [file antibiotics-09-00274-s001.pdf]

SUPPLEMENTARY MATERIALS

1. Fungal

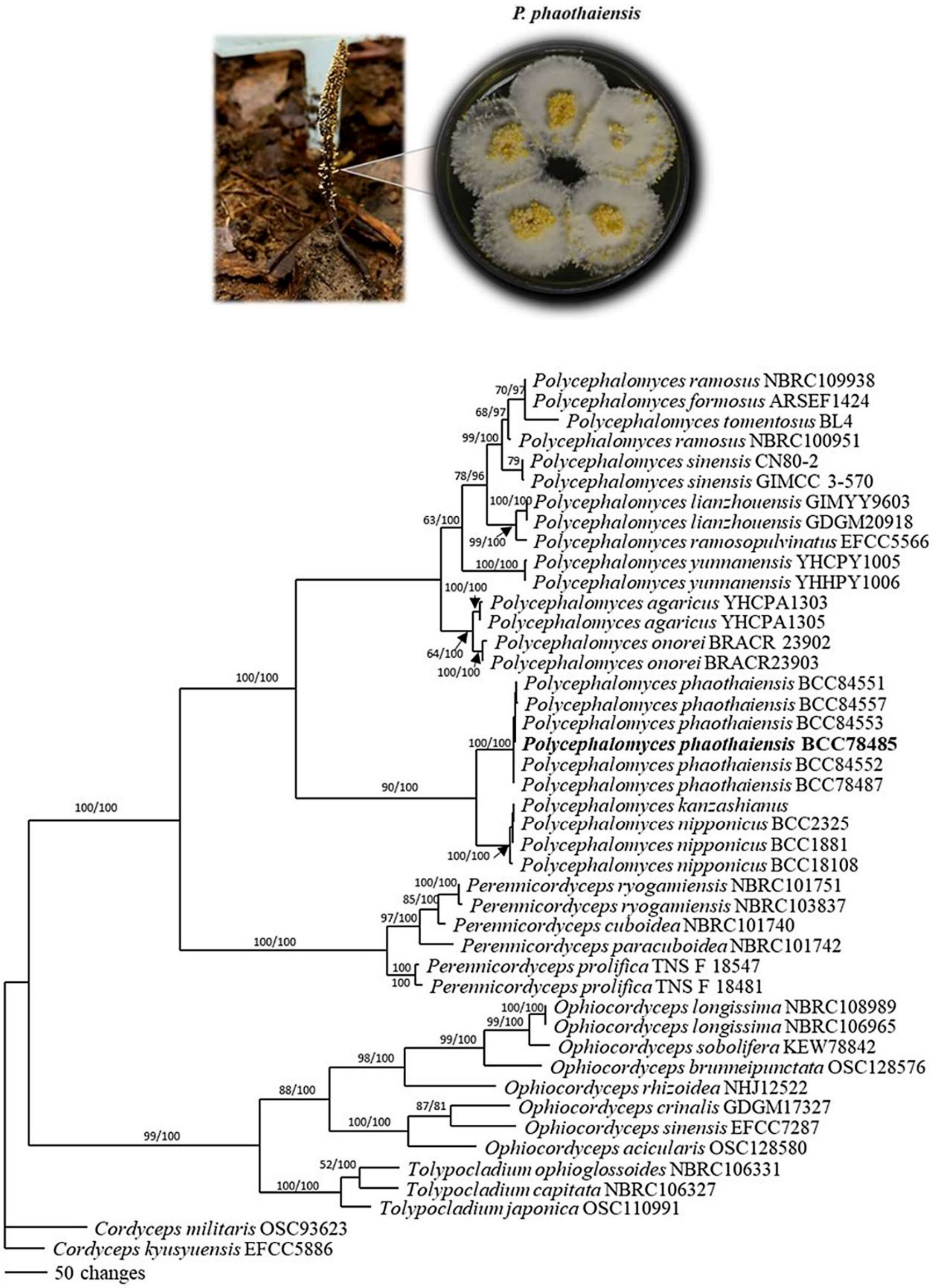

Figure S1 Colony appearance and phylogenetic tree of *P. phaothaiensis* BCC78485

2. Isolated compounds

Cordytropolone (**1**): colorless solid, $C_9H_8O_4$, ESI-MS (neg. ion mode) *m/z* 179.037 $[M-H]^-$; FT-IR (ATR) ν_{max} (cm^{-1}): 3188, 1603, 1520, 1434, 1377, 1275, 1219, 1171, 917, 762, 710.

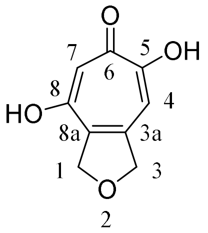

Table S1. NMR data of **1** (in DMSO-d_6) measured at 400 (^{1}H) and 100 (^{13}C) MHz

Position	δ_C (ppm)	δ_H (ppm), mult (*J* in Hz)	^{1}H-^{1}H COSY	HMBC
1	75.1	4.95 *t* (2.84, 3.16)	3	3, 3a, 4, 7, 8, 8a
3	77.9	5.02, *t* (2.96, 3.12)	1, 4	1, 3a, 4, 5, 8, 8a
3a	145.6	-	-	-
4	106.6	6.81, *s*	3	3, 3a, 5, 6, 8a
5	164.4	-	-	-
6	173.1	-	-	-
7	113.2	6.79, *s*	-	1, 3a, 5, 6, 8, 8a
8	162.1	-	-	-
8a	127.5	-	-	-

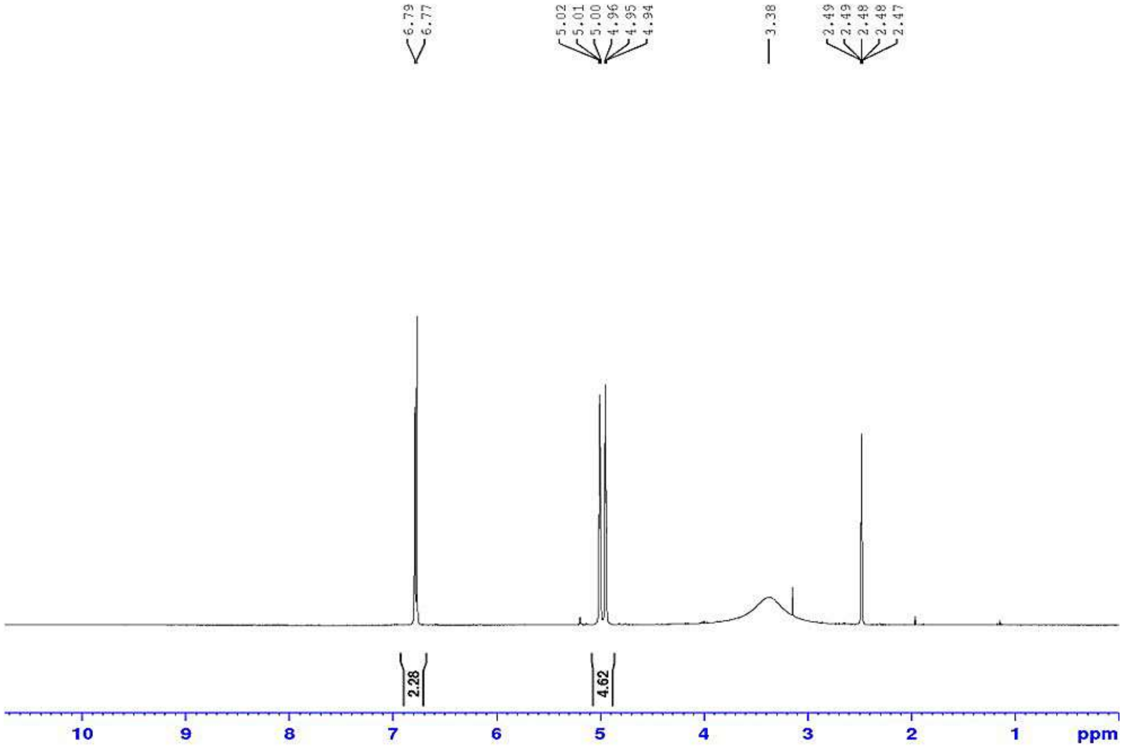

Figure S2 ^{1}H-NMR spectrum of **1** (400 MHz, DMSO-d_6)

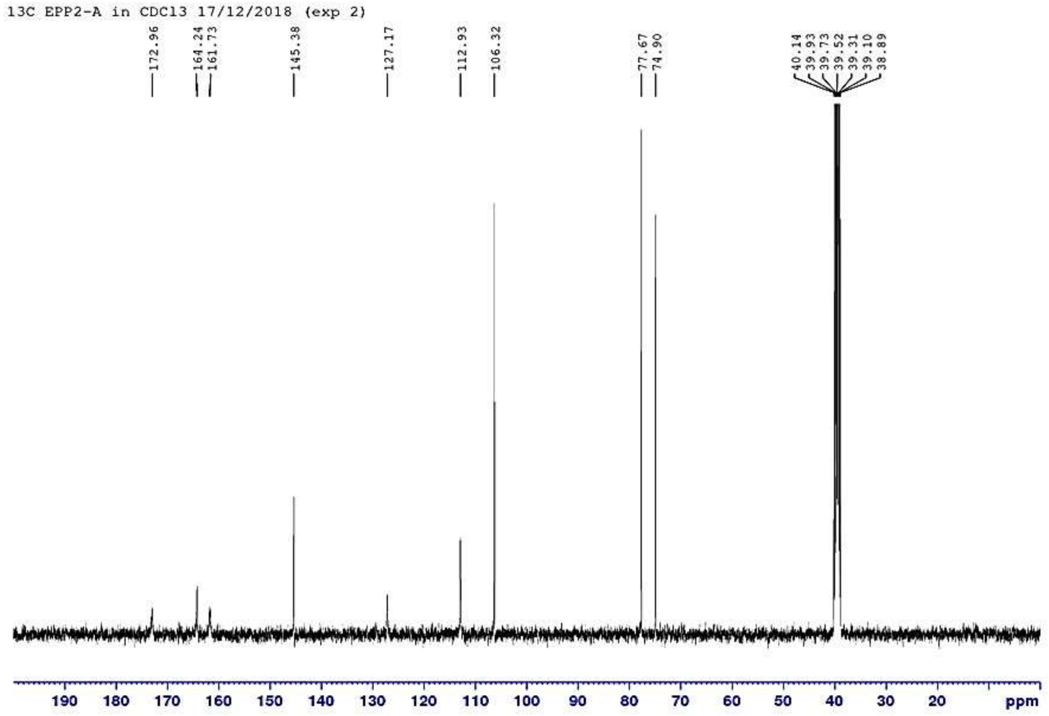

Figure S3 ^{13}C-NMR spectrum of **1** (100 MHz, DMSO-d_6)

Stipitalide **(2)**: reddish-brown amorphous, $C_9H_7O_5$, ESI-MS (pos. ion mode) *m/z* 195.0292 $[M+H]^+$, FT-IR (ATR) ν_{max} (cm^{-1}): 3146, 1716, 1628, 1482, 1228, 1168, 1048, 1028. ^{1}H-NMR (DMSO-d_6) δ (ppm): 6.88 (1H, *s*, H-7), 6.82 (1H, *s*, H-3), 5.25 (2H, *s*, H-9), ^{13}C-NMR (DMSO-d_6) δ (ppm):172.4 (C-1), 171.1 (C-8), 170.2 (C-2), 164.9 (C-4), 157.9 (C-6), 112.3 (C-3), 109.8 (C-5), 108.7 (C-7), 70.1 (C-9).

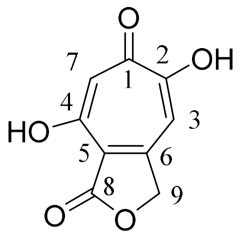

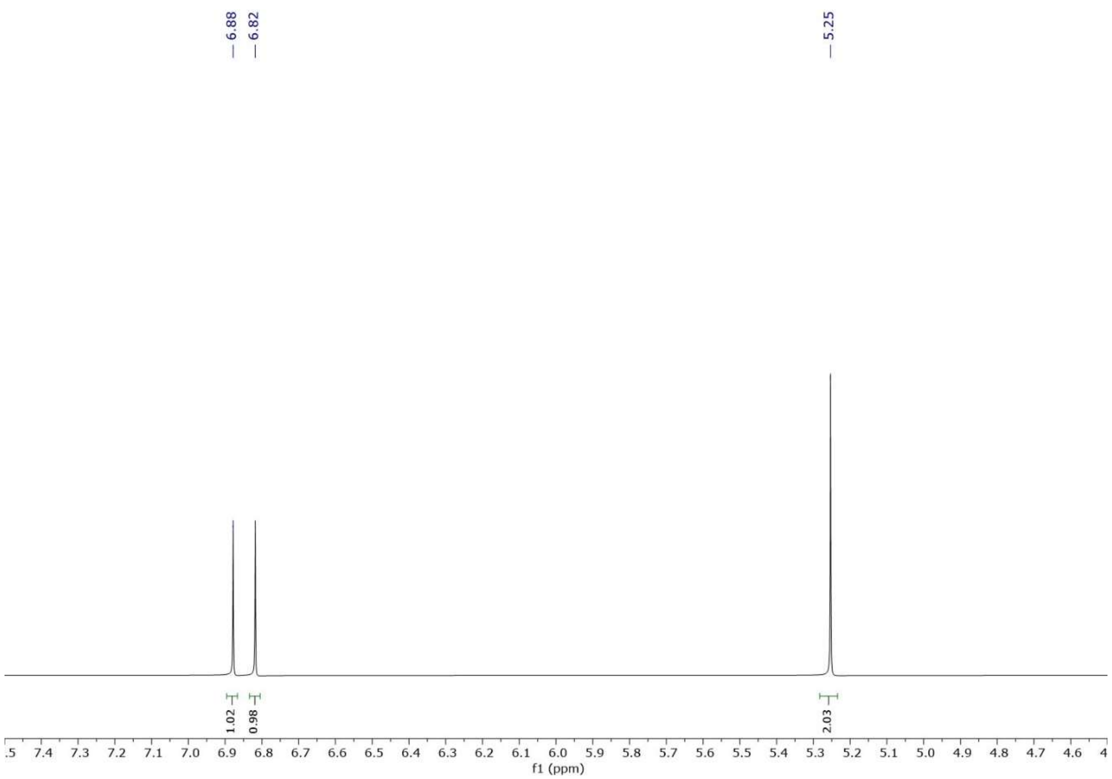

Figure S4 ^{1}H-NMR spectrum of **2** (400 MHz, DMSO-d_6)

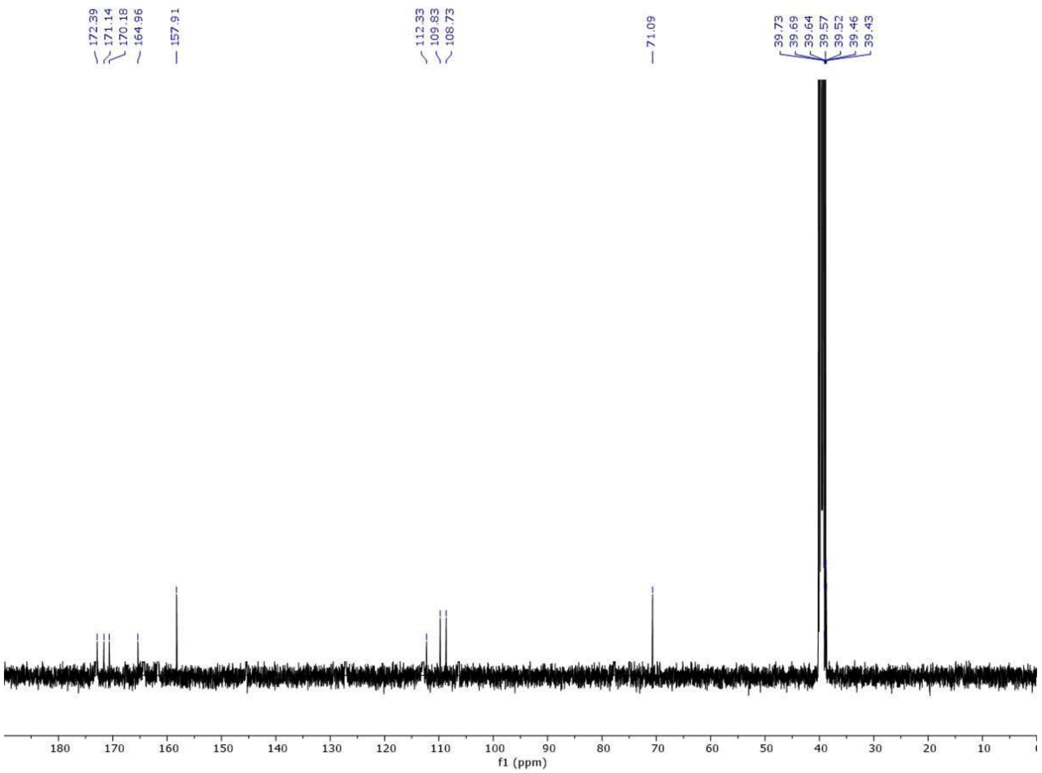

Figure S5 ^{13}C-NMR spectrum of **2** (100 MHz, DMSO-d_6)

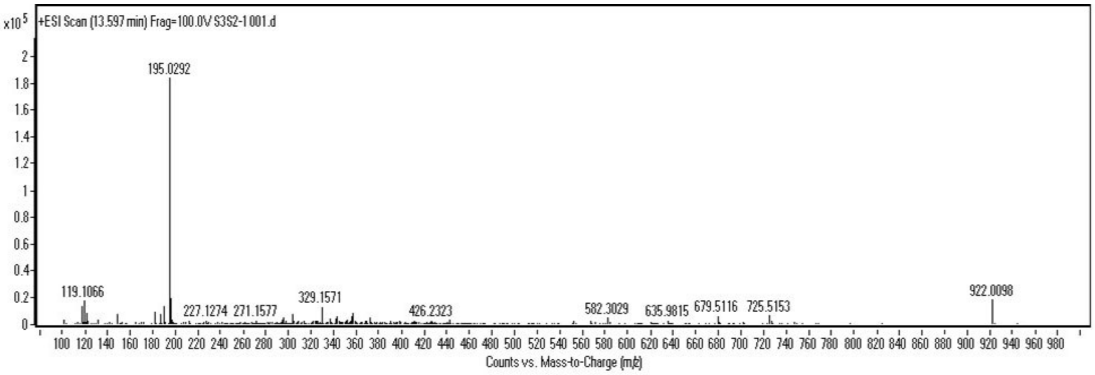

Figure S6 ESI-MS (pos. ion mode) spectrum of **2**

(+)-Piliformic acid or (*R*,*E*)-2-hexylidene-3-methylsuccinic acid **(3)**: white solid, $C_{11}H_{18}O_4$; ESI-MS (neg. ion mode) *m/z* 213.119 [M-H]⁻; $[\alpha]_D^{28}$ +1.3 (MeOH, *c* 0.155); UV (MeOH) λ_{max} (log ε) 241 nm (2.67); FT-IR (ATR) ν_{max} (cm^{-1}): 3250, 2926, 2857, 1684, 1626, 1410, 1292, 1256, 1223, 1058.

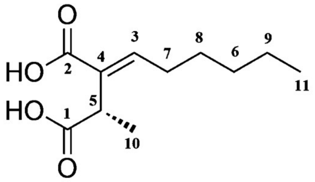

Table S2
NMR data of **3** (in $CDCl_3$) measured at 400 (^{1}H) and 100 (^{13}C) MHz

Position	δ_C (ppm)	δ_H (ppm), mult (*J* in Hz)	^{1}H-^{1}H COSY	HMBC
1	180.2			
2	172.1			
3	147.5	7.02, *t* (7.6)	7	2, 4, 5, 7, 8, 10
4	131.2			
5	37.6	3.67, *q* (7.1, 1.5)	10	1, 2, 3, 4, 10
6	31.6	1.24-1.34, *m*	8, 9	7, 8, 9, 11
7	28.9	2.34-2.13, *m*	8	2, 3, 4, 6, 8
8	28.2	1.48, *m*	6, 7	3, 6, 7, 9
9	22.5	1.24-1.34, *m*	6, 11	6, 8, 11
10	15.6	1.36, *d* (7.1)	5	1, 4, 5
11	14.0	0.89, *m*	9	6, 9

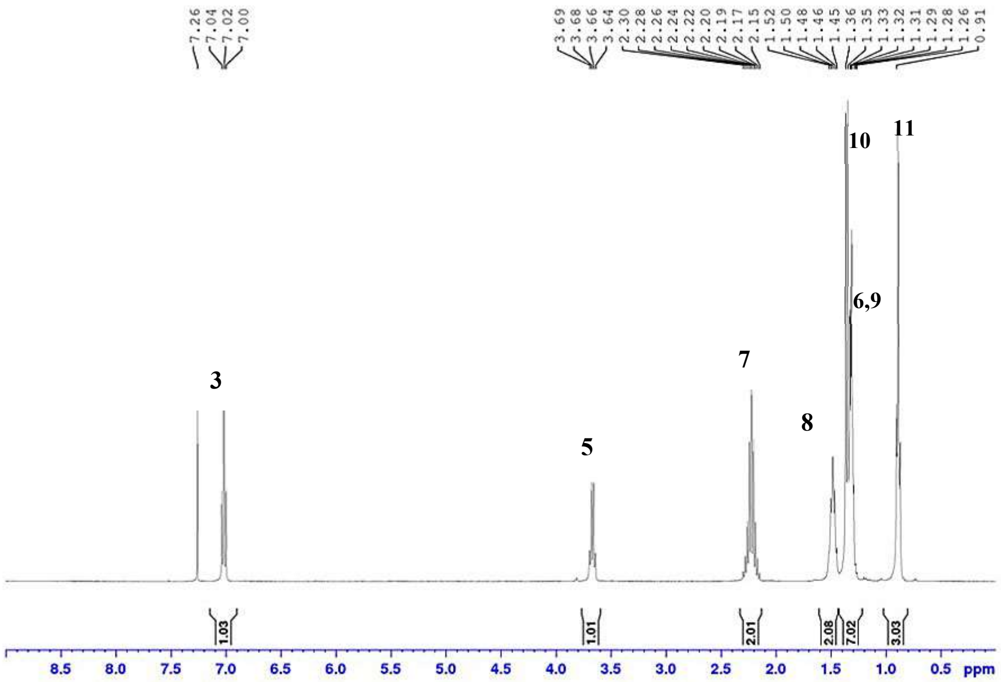

Figure S7 ^{1}H-NMR spectrum of **3** (400 MHz, $CDCl_3$)

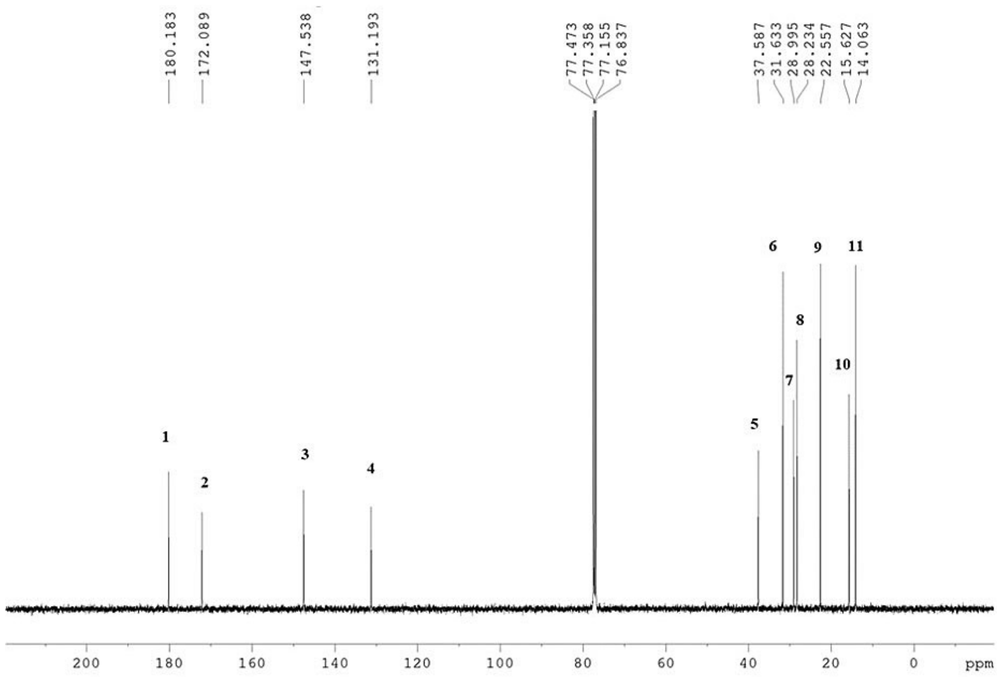

Figure S8 ^{13}C-NMR spectrum of **3** (400 MHz, $CDCl_3$)

D-manitol **(4)**: white solid, $C_6H_{14}O_6$, ESI-MS (pos. ion mode) *m/z* 205.0679 $[M+Na]^+$; FT-IR (ATR) ν_{max} (cm^{-1}): 3234, 2875, 1635, 1411, 1363, 1062, 1032, 985. ^1H-NMR (D_2O) δ (ppm): 3.88 (1H, *dd*, *J* = 11.7, 2.6 Hz, H-1a), 3.82 (1H, *d*, *J* = 8 Hz, H-3), 3.77 (1H, *m*, H-2), 3.69 (1H, *dd*, *J* = 11.6, 5.9 Hz, H-1b).

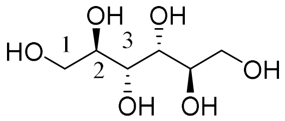

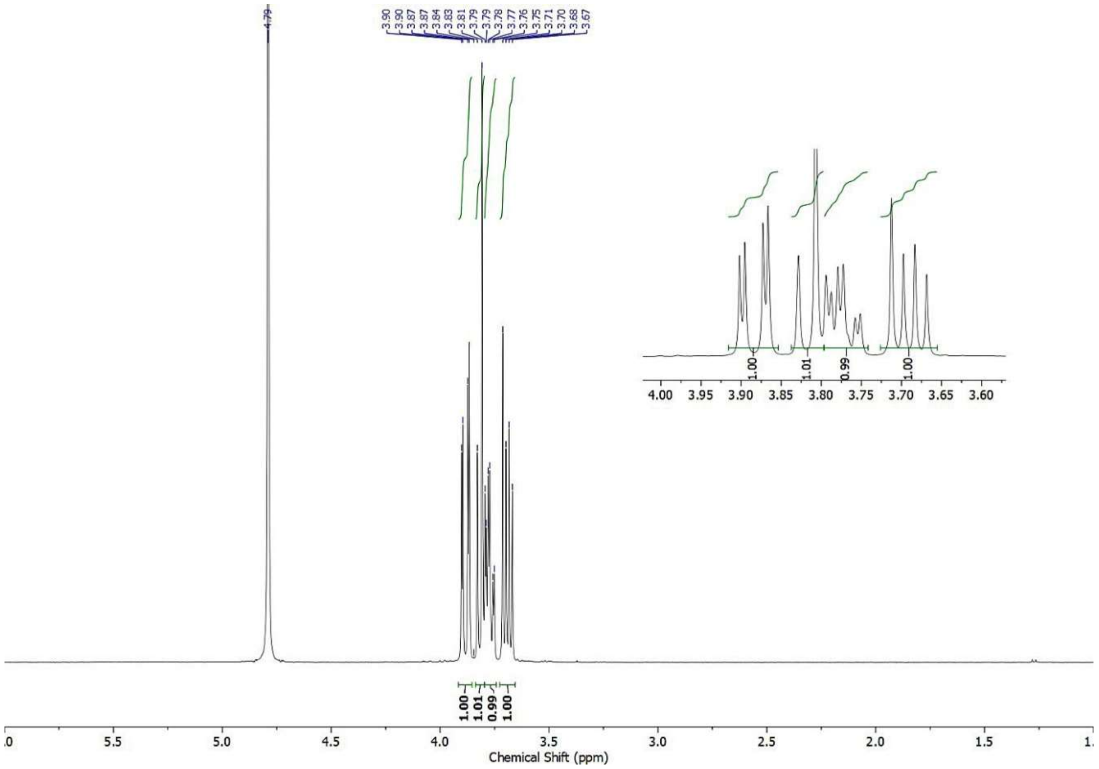

Figure S9 ^1H-NMR spectrum of **4** (400 MHz, D_2O)

Methyl linoleate **(5)**: colorless oil, $C_{19}H_{34}O_2$, EI-MS *m/z* 294 $[M]^+$ ^{1}H-NMR (CDC1$_3$) δ (ppm): 5.42-5.27 (4H, *m,* H-9, H-10, H-12, H-13), 2.77 (2H, *t*, *J*= 6.6 Hz, H-11), 2.29 (2H, *dt*, *J*= 9.7, 7.5 Hz, H-2), 2.03 (8H, *dq*, *J*= 15.2, 6.8 Hz, H-8, H-14), 1.62 (2H, *t*, *J*= 6.9 Hz, H-3), 1.57 (3H, *s*, H-1′), 1.39-1.20 (14H, *m*, H-4, H-5, H-6, H-7, H-15, H-16, H-17), 1.09-0.76 (3H, *m*, H-18). A mass spectrum of **5** was also determined by GC-MS computer matching with Wiley7n library (99% matching).

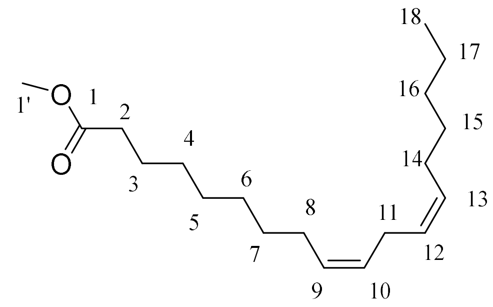

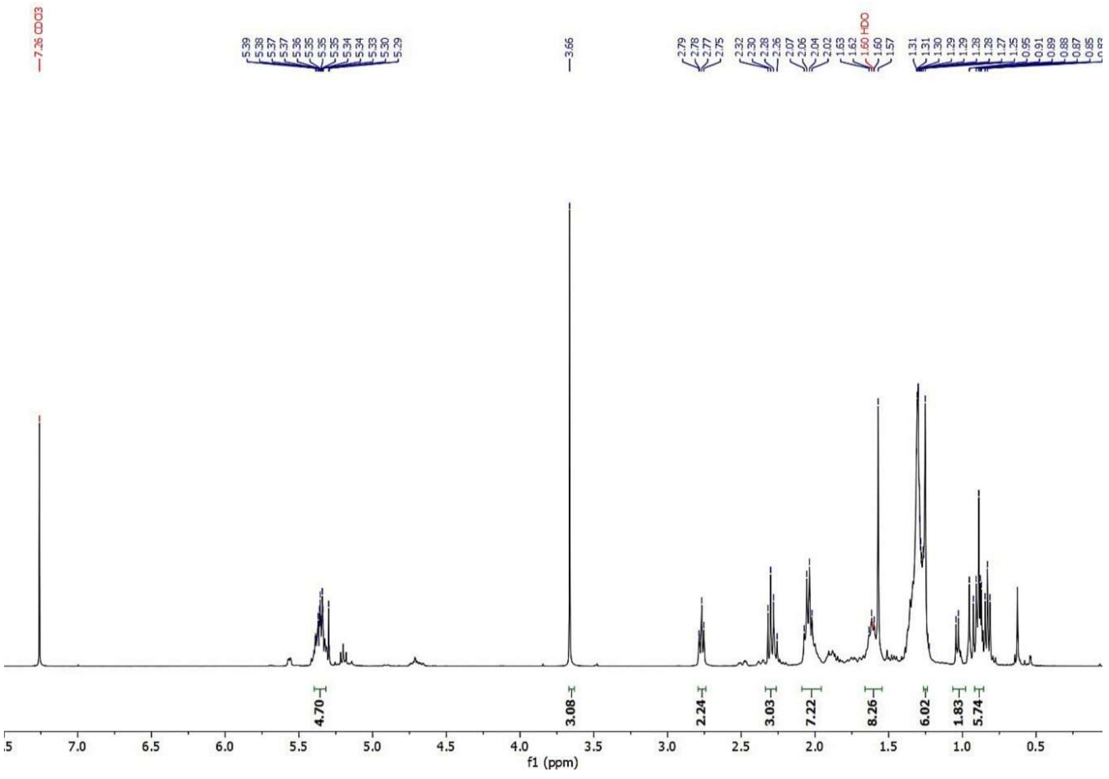

Figure S10 ^{1}H-NMR spectrum of **5** (400 MHz, $CDCl_3$)

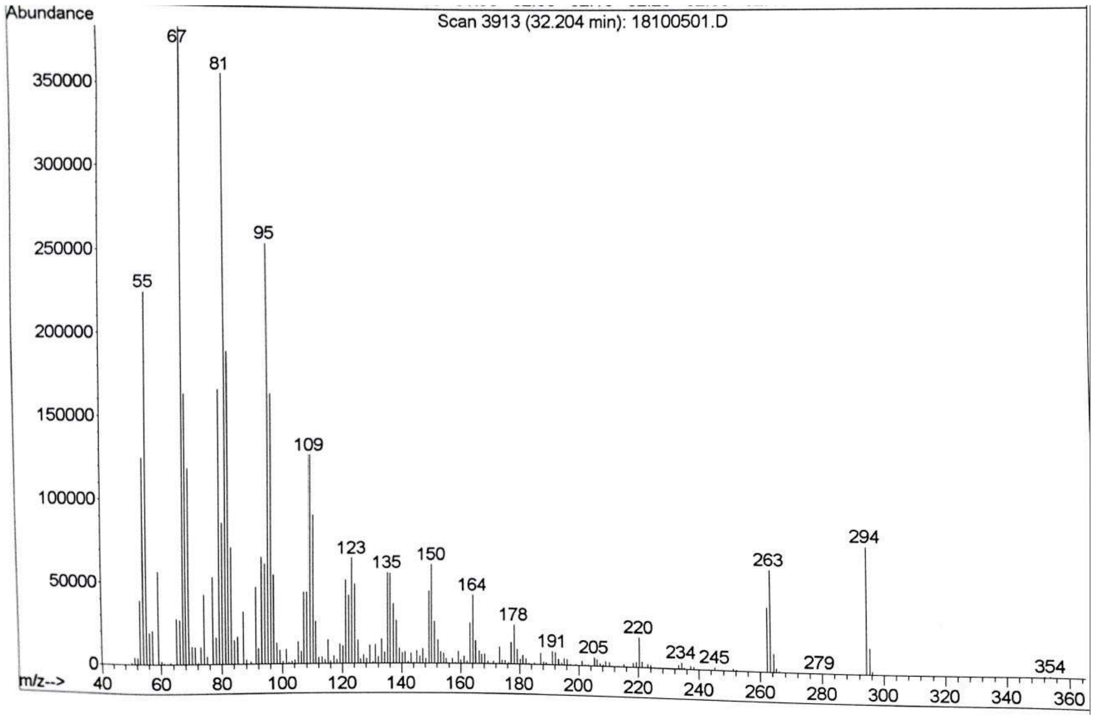

Figure S11 EI-MS spectrum of **5**

Linoleic acid **(6)**: colorless oil, $C_{18}H_{32}O_2$, EI-MS *m/z* 280 $[M]^+$. ^{1}H-NMR ($CDC1_3$) δ (ppm): 5.36 (4H, *qt*, *J* = 8.4, 4.3 Hz, H-9, 10, 12, 13), 2.77 (2H, *t*, *J* = 6.3 Hz, H-11), 2.35 (2H, *t*, *J* = 7.5 Hz, H-2), 2.04 (8H, *p*, *J* = 7.9, 7.3 Hz, H-8, 14), 1.63 (2H, *p*, *J* = 7.4 Hz, H-3), 1.29 (14H, *m*, H-4, 5, 6, 7, 15, 16, 17), 0.88 (3H, *td*, *J* = 6.7, 6.0, 3.2 Hz, H-18). A mass spectrum of **6** was also determined by GC-MS computer matching with Wiley7n library (96% match).

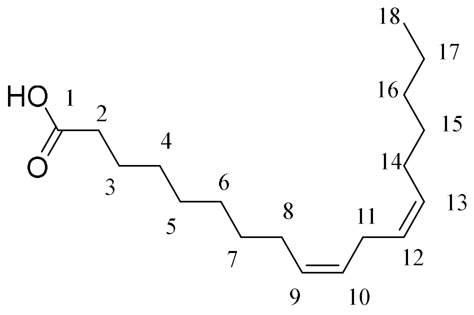

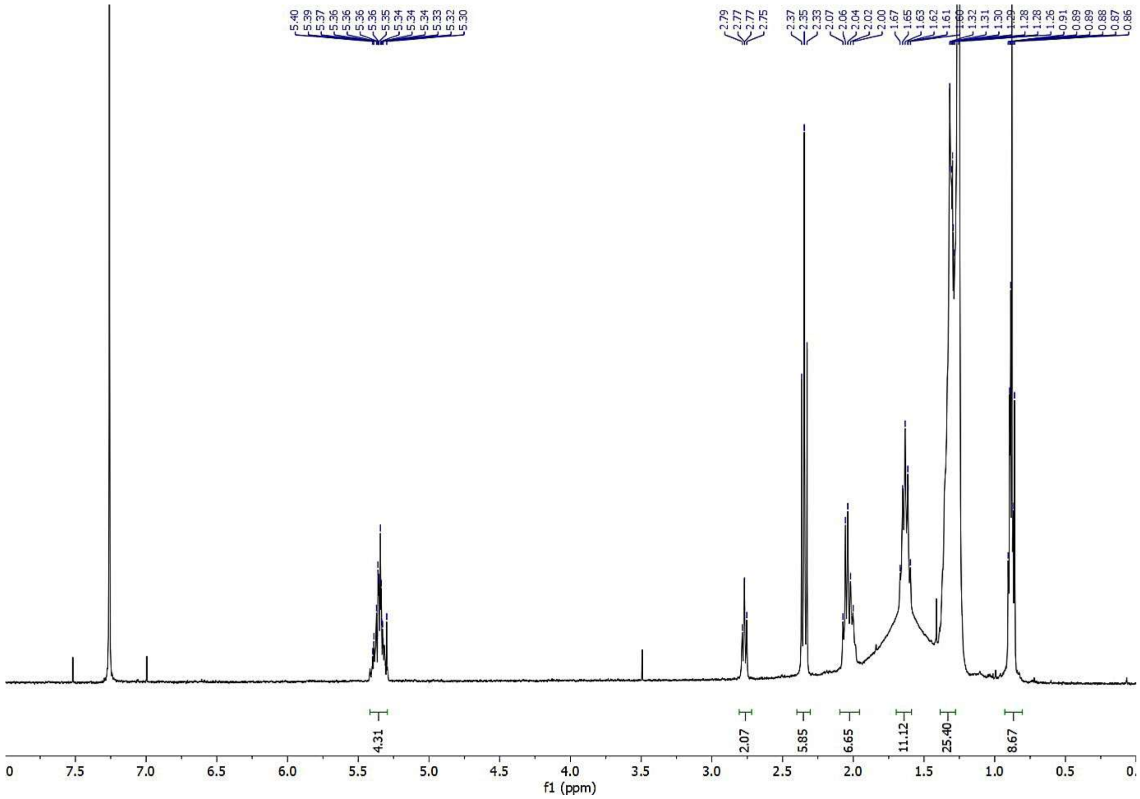

Figure S12 ^{1}H-NMR spectrum of **6** (400 MHz, $CDCl_3$)

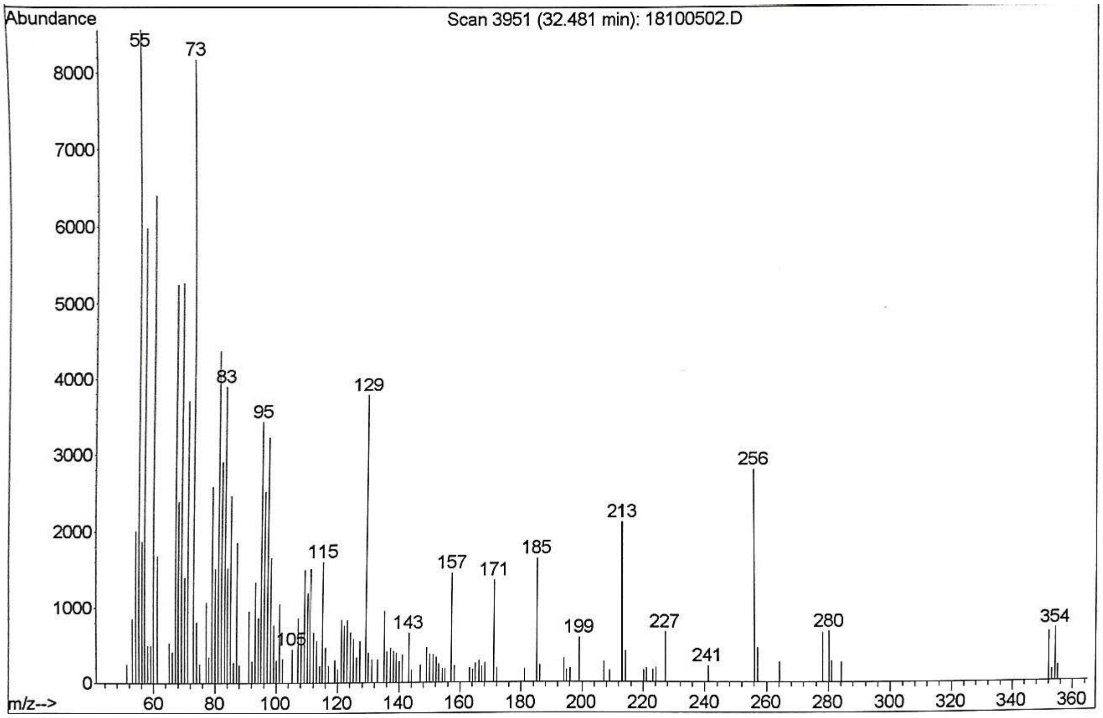

Figure S13 EI-MS spectrum of **6**

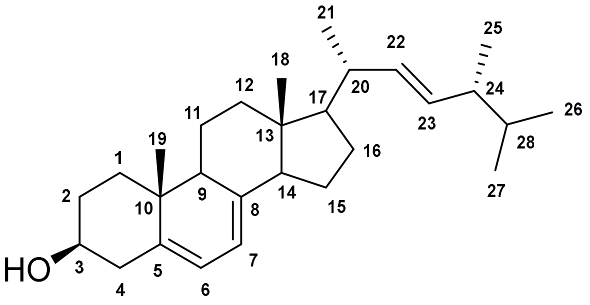

Ergosterol **(7)** Colorless solid, $C_{28}H_{44}O$, ESI-MS (pos. ion mode) *m/z* 396.65 $[M+H]^+$; FT-IR (ATR) ν_{max} (cm^{-1}): 3413, 2952, 2869, 1738, 1656, 1457, 1366, 1326, 1239, 1053, 1031, 967, 802. ^{1}H-NMR (CDC1$_3$) δ (ppm): 5.57(1H, *dd*, *J* = 5.8, 2.6 Hz, H-6), 5.38(1H, *dt*, *J* = 5.6, 2.8 Hz, H-8), 5.28–5.12 (2H, *m*, H-22, H-23), 3.69–3.58 (1H, m, H-3). The position of this signal varied from 1.20–2.50 ppm in the other saturated methylene and methine protons (total 21H); 1.03 (3H, d, *J* = 6.7 Hz, H-21), 0.95 (3H, *s*, H-19), 0.91, 0.93 (3H, *d*, *J* = 6.8 Hz, H-28), 0.83, 0.85 (3H, *d*, J = 6.7 Hz, H-26), 0.81, 0.84 (3H, d, J = 6.7 Hz, H-27), 0.63 (3H, S, H-18).

^{13}C-NMR (CDC1$_3$) δ (ppm):141.5 (C-8), 139.9 (C-5), 135.7 (C-22), 132.1 (C-23), 119.7 (C-6), 116.4 (C-7), 70.6 (C-3), 55.8 (C-17), 54.7 (C-14), 46.4 (C-9), 42.9 (C-13), 42.9 (C-24), 40.9 (C-4), 40.5 (C-20), 39.2 (C-12), 38.5 (C-1), 37.1 (C-10), 33.2 (C-25), 32.1 (C-2), 28.4 (C-16), 23.1 (C-15), 21.2 (C-21), 20.1 (C-11), 19.8 (C-27), 19.6 (C-26), 17.7 (C-19), 16.4 (C-28), 12.2 (C-18)

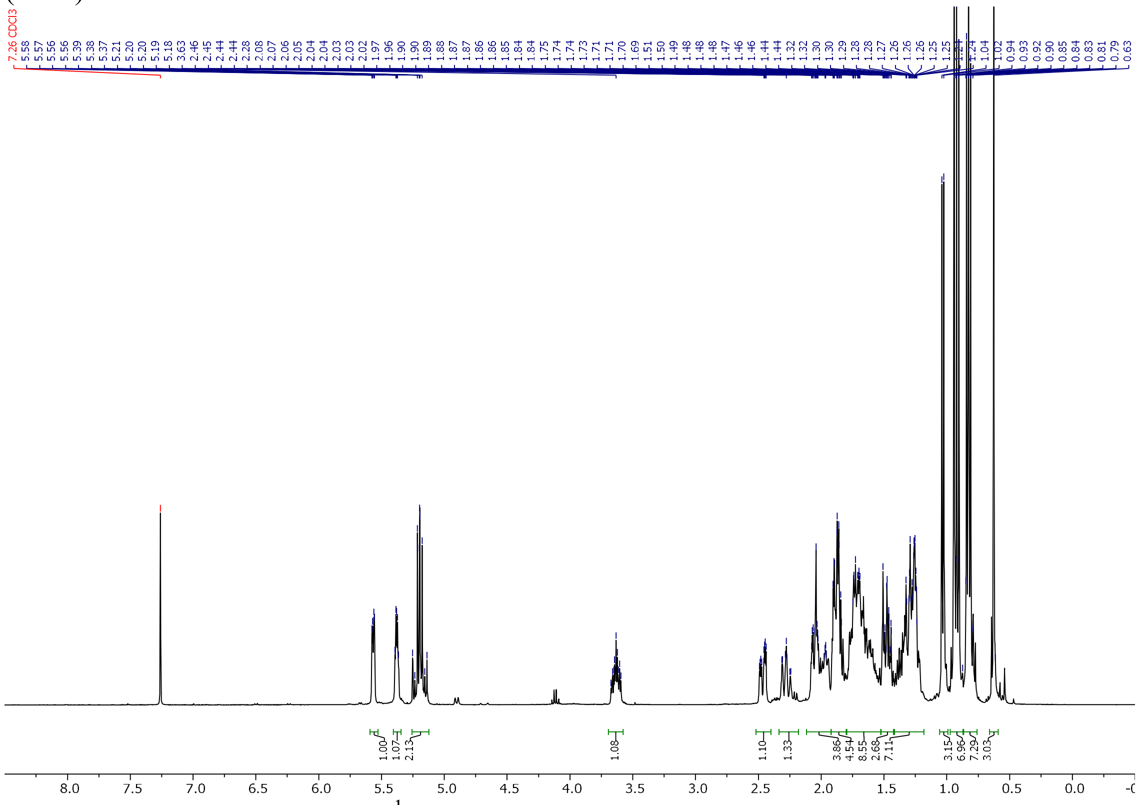

Figure S14 ^{1}H-NMR spectrum of **7** (400 MHz, $CDCl_3$)

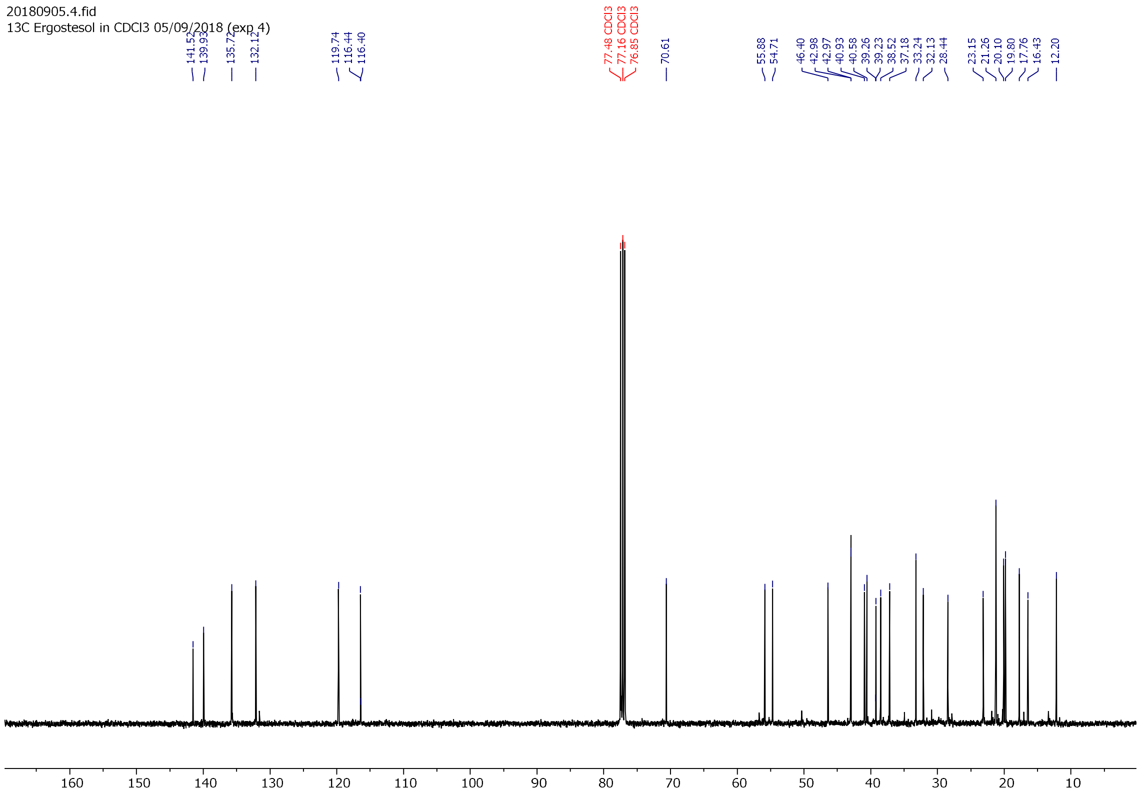

Figure S15 ^{13}C-NMR spectrum of **7** (100 MHz, $CDCl_3$)

3. Antibacterial activity

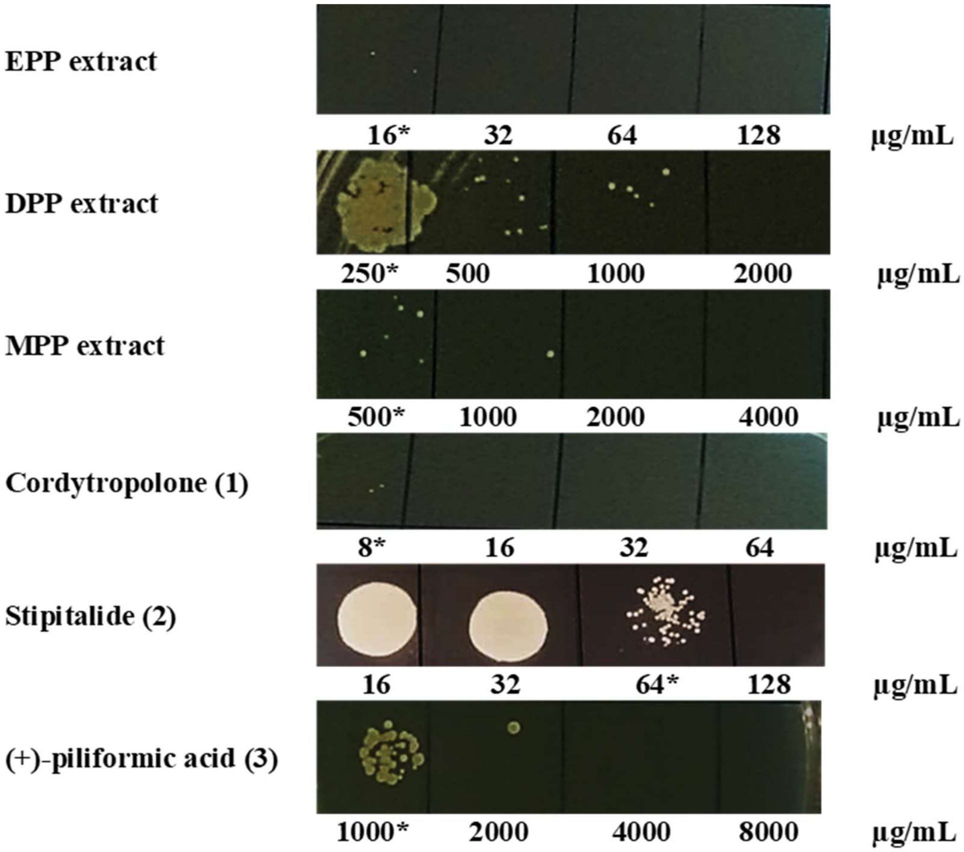

Figure S16 Minimum bactericidal concentration (MBC) test of extracts, cordytropolone **(1)**, stipitalide **(2)**, and (+)-piliformic acid **(3)** from *P. phaothaiensis* against *P. acnes* **(A)**. *concentration at MIC values
